# Antibacterial Ability of Sodium Hypochlorite Heated in the Canals of Infected Teeth: An Ex Vivo Study

**DOI:** 10.7759/cureus.6975

**Published:** 2020-02-13

**Authors:** Ghassan Yared, Ghada Al Asmar Ramli

**Affiliations:** 1 Endodontist, Private Practice, Beirut, LBN; 2 Orthodontics, Faculty of Dental Medicine, Lebanese University, Beirut, LBN

**Keywords:** intracanal heating of sodium hypochlorite, endoactivator, eddy, passive ultrasonic activation, root canal bacteria elimination

## Abstract

Background

Apical periodontitis is caused by bacteria present in the root canal space. The removal of the infection is crucial to obtain healing. Canal irrigation is among one of the most important steps in eliminating bacteria. Sodium hypochlorite (NaOCl) is still the preferred irrigant due to its disinfecting and pulpal dissolution abilities. Heating NaOCl improves those abilities. However, the ability of intracanal heated NaOCl to kill bacteria has not yet been evaluated.

Objectives

This study compared the disinfecting ability of different irrigation regimens using NaOCl with and without sonic and ultrasonic agitation, and with and without intracanal heating of NaOCl.

Methods

The canals of extracted mandibular premolars were prepared, sterilized and infected with E. faecalis for 28 days. The canals were then assigned to eight groups of 10 teeth depending on the NaOCl irrigation protocol. Group CONV: conventional irrigation with syringe and needle; Group END: NaOCl sonic agitation with EndoActivator; Group EDD: NaOCl sonic agitation with EDDY; Group PUI: NaOCl passive ultrasonic agitation; Group H: intracanal heating of NaOCl; Groups END-H, EDD-H and PUI-H: NaOCl agitation with EndoActivator, EDDY and passive ultrasound, respectively, followed by intracanal heating of NaOCl. The canals were sampled before (S1) and after (S2) the different irrigation protocols were performed, the colony-forming units were counted and the percentage of bacteria reduction was calculated for each group.

Results

The number of bacteria decreased significantly for the different protocols (p < 0.001). The groups with NaOCl intracanal heating reduced bacteria significantly more than the other groups (p < 0.001). Five S2 samples in group H were free of bacteria. All of the S2 samples in the groups with NaOCl sonic and ultrasonic agitation followed by NaOCl heating were free of bacteria. Intracanal heating of NaOCl was more effective in killing bacteria than conventional irrigation, and sonic or passive ultrasonic agitation of NaOCl.

Conclusions

Intracanal heating of NaOCl has the potential to be used as an adjunct to root canal irrigation in order to increase bacterial reduction in comparison to the conventional irrigation techniques involving sonic or ultrasonic agitation. Agitation of NaOCl followed by intracanal warming of the solution seems to be very promising in eliminating bacteria from infected root canals.

## Introduction

Apical periodontitis is mainly caused by bacteria present in the root canal space [1]. One of the main objectives of a root canal treatment is the removal of bacteria [2]. Root canal irrigation plays a crucial role in eliminating bacteria and sodium hypochlorite (NaOCl) is still the preferred irrigant solution due to its increased disinfecting and pulp tissue dissolving abilities [3].

Heating NaOCl increases its dissolving and disinfecting properties [4-7]. Warming of NaOCl can be done by either preheating the solution outside of the canal or heating it inside the canal [4,8]. However, intracanal heating of NaOCl was more effective than pre-heating with regards to canal cleanliness and presence of debris [9]. Moreover, ultrasonic agitation of intracanal heated NaOCl resulted in cleaner canals compared to conventional and passive ultrasonic irrigation [10].

Canal cleanliness and/or the presence of debris have not been shown to influence the repair of tissues [11]. Indirect evidence suggested that a higher degree of canal cleanliness would not affect outcome [12]. On the other hand, the presence of bacteria and its adverse influence on endodontic outcome, and the importance to remove infection to prevent disease or obtain healing have been well established [13]. Therefore, the objective of the present study was to evaluate the ability of NaOCl heated in infected canals of extracted mandibular premolars to kill bacteria. More specifically, this study compared the antibacterial activity of different irrigation regimens with NaOCl: conventional irrigation, sonic agitation, passive ultrasonic irrigation, intracanal heating of NaOCl, and sonic and passive ultrasonic NaOCl agitation followed by intracanal heating of NaOCl.

## Materials and methods

Tooth selection and preparation

Eighty-eight single rooted mandibular premolars previously extracted for orthodontic reasons and stored in 0.9% saline solution were selected for this ex vivo study. None of the teeth had an endodontic treatment. The roots were fully developed. All teeth had only one canal, and did not show root fracture, resorptive defects or canal calcifications. The root surfaces were cleaned with periodontal curettes. The teeth were re-placed in a 0.9% saline solution until use.

The access cavity was created. Patency was established using a #8 hand file (Maillefer, Ballaigues, Switzerland) inserted in the canal until its tip was visible at the apical foramen. The crown was then reduced to standardize the length of the teeth. The working length (WL) was established a 17 mm, 1 mm shorter than the apical foramen. All canals were instrumented to working length with Reciproc 25 (0.25 mm at the tip) (VDW GmbH, Munich, Germany) followed by a Reciproc 50 (0.50 mm at the tip) (VDW GmbH). The instruments were used according to the manufacturer’s instructions for use. The canals were irrigated during the preparation procedure with 6 ml of 5.25% NaOCl using a syringe and a NaviTip 31-ga endodontic irrigation needle with double sideport (Ultradent Products Inc., South Jordan, UT). The final irrigation protocol consisted of 3 ml of 17% ethylenediaminetetraacetic acid (EDTA) (Ultradent Products Inc.), 3 ml of distilled water and 3 ml of 5.25% NaOCl, each for 2 minutes. The NaOCl was inactivated with 3 ml 10% sodium biosulfate for 2 minutes. Sterile Reciproc 50 paper points (VDW GmbH) were used to dry the canals. The root surfaces and the apex of each tooth were sealed with cyanoacrylate. The teeth were mounted in silicone blocks. The specimens were distributed into eight groups of 11 specimens each. All the specimens were then sterilized at 121°C for 30 minutes.

Control of sterilization

One canal from each group was selected randomly to serve as a negative control and confirm sterilization. It was incubated and cultured in the same manner as described later for the root canal sampling procedure.

Specimen contamination

E. faecalis, derived from ATCC 19433, was used and cultured aerobically on blood agar at 35°C for 48 h. Inoculum was prepared in sterile BHI broth and turbidity was set to 0.5 McFarland corresponding to approximately 1.5 × 10^8^ colony forming units per milliliter (CFU/mL).

Ten canals from each group were inoculated with 10 μL of the E. faecalis culture. The specimens were incubated for 28 days at 37°C. The inoculum was refreshed every day.

Experimental groups

Each experimental group included 10 specimens. The eight groups were as follows:

1- Group CONV (n = 10): Each canal was irrigated with room temperature 5.25% NaOCl for 3 minutes using a syringe and a NaviTip 31-gauge endodontic irrigation needle with double sideport (Ultradent Products Inc.). A sterile stopper was placed on the needle to verify its insertion to 1 mm short of the working length. The needle was moved up and down over 3-5 mm during the irrigation. Each canal was rinsed with 9 mL of 5.25% NaOCl.

2- Group END (n = 10): Each canal was filled with room temperature 5.25% NaOCl. The EndoActivator sonic device (Dentsply, Ballaigues, Switzerland) was used to agitate the solution in the canal with a specifically designed tip according to the manufacturer’s recommendations [14]. The tip was introduced to 1 mm less than the WL. The solution agitation with the EndoActivator tip was repeated three times. The canal was flushed with 3 mL room temperature 5.25% NaOCl after each activation cycle to refresh the solution.

3- Group EDD (n = 10): Each canal was filled with room temperature 5.25% NaOCl. The EDDY sonic device (VDW GmbH) was used to agitate the solution in the canal with a specifically designed tip according to the manufacturer’s recommendations [15]. The tip was introduced to 1 mm less than the WL. The solution agitation with the EDDY tip was repeated three times. The canal was flushed with 3 mL room temperature 5.25% NaOCl after each activation cycle to refresh the solution.

4- Group PUI (n = 10): Each canal was filled with room temperature 5.25% NaOCl. An ultrasonic tip with a noncutting end (Irrisafe tip K20/21 mm; Acteon, Mt Laurel, NJ) mounted in a piezoelectric ultrasonic device (P5; Satelec Acteon, Merignac, France) was inserted to 1 mm less than the WL and activated at the power setting of 4 for 20 seconds. Passive ultrasonic agitation (PUI) was repeated three times. The canal was flushed with 3 mL room temperature 5.25% NaOCl after each activation cycle to refresh the solution [16].

5- Group H (n = 10): Each canal was filled with room temperature 5.25% NaOCl. The solution was heated in the canal for 10 s using a Touch’n Heat XF (size 0.30 mm and 0.04 mm/mm taper) electric heat carrier (Analytic Endodontics, Orange, CA), attached to a System B unit (Analytic Endodontics) [17]. The temperature was set at 150°C. The heat carrier was inserted to 1 mm short of the working length. This procedure was repeated three times. The canal was flushed with 3 mL room temperature 5.25% NaOCl after each activation cycle to refresh the solution. During the heating procedure, the heat carrier was moved with small, in- and out-movements in the canal. Care was taken to avoid wedging the heat carrier in the canal.

6- Group END-H (n = 10): Sonic agitation was performed three times as described for group END. After each time, NaOCl was aspirated, the canal was filled again with room temperature 5.25% NaOCl which was heated in the canal in the same manner as described for group H. Three cycles of heating were performed as for group H.

7- Group EDD-H (n = 10): Sonic agitation was performed three times as described for group EDDY. After each time, NaOCl was aspirated, the canal was filled again with room temperature 5.25% NaOCl which was heated in the canal in the same manner as described for group H. Three cycles of heating were performed as for group H.

8- Group PUI-H (n = 10): Passive ultrasonic agitation was performed three times as described for group PUI. After each time, NaOCl was aspirated, the canal was filled again with room temperature 5.25% NaOCl which was heated in the canal in the same manner as described for group H. Three cycles of heating were performed as for group H.

At the end of each procedure, the NaOCl was then inactivated with 3 mL of 10% sodium biosulfate for 2 minutes.

All solutions were delivered into the canal using a 3-mL syringe and a NaviTip 31-gauge endodontic irrigation needle with double sideport (Ultradent Products Inc.).

Root canal sampling procedures

Ten canals from each group were sampled.

A first sample (S1) was taken from each canal prior to implementing the tested protocols. The canal was filled with a sterile saline solution. A #15 hand file (Maillefer) was used to loosen biofilm bacteria by scraping the root canal walls for 15 s. A sterile Reciproc 50 paper point was inserted in the canal to the working length. The paper point was left in the canal for 1 minute and placed in sterile BHI broth for 20 minutes. The solution was homogenized by vortex, serially diluted, plated on blood agar and incubated for 48 h at 37°C. Colony-forming units (CFUs) were counted.

A second sample (S2) was taken from each canal after implementing the investigated irrigation protocol. The number of CFUs was determined as described for S1.

Statistical analysis

The Statistical Package Software for Social Science (SPSS for Windows, Version 22.0, Chicago, IL, USA) was used to perform the statistical analysis. The level of significance was set at p ≤ 0.05. Kolmogorov-Smirnov tests were conducted to evaluate the normality distribution of continuous variables. Wilcoxon tests were performed to compare colony-forming units before and after irrigation for the eight groups. The percentage of variation of CFU after irrigation was compared among the eight groups using Kruskal-Wallis tests, followed by Mann-Whitney tests.

## Results

The results are shown in Table 1.

**Table 1:** Mean and standard deviation of CFUs in each group, for S1, S2 and the percentage of difference in CFUs between S2 and S1. *Sample taken before (S1) and after (S2) irrigation. †Number of colony-forming units was significantly less after irrigation compared to before irrigation for the different groups (p < 0.001). ‡Percentage of S2-S1: percentage of bacteria reduction. Different superscript letters (a, b and c) indicate statistical difference. ††CON: Conventional irrigation; EDD: Agitation of NaOCl with EDDY sonic device; END: Agitation of NaOCl with EndoActivator sonic device; PUI: Passive ultrasonic agitation of NaOCl; HEAT: Intracanal heating of NaOCl; EDD+HEAT: Sonic agitation of NaOC with EDDY device followed by intracanal heating of NaOCl; END+HEAT: Sonic agitation of NaOC with EndoActivator device followed by intracanal heating of NaOC; PUI+HEAT: Passive ultrasonic agitation of NaOC followed by intracanal heating of NaOCl.

Irrigation protocols^††^	S1*^†^	S2*^†^	Percentage of S2-S1^‡^
CONV	8.53 x 10^8^ ± 3.6 x 10^8^	10.03 x 10^6^ ± 5.6 x 10^6^	98.824 ± 0.421^a^
EDD	1.11 x 10^9 ^± 2.9 x 10^8^	6.07 x 10^5 ^± 5.8 x 10^5^	99.942 ± 0.059^b^
END	8.29 x 10^8^ ± 2.6 x 10^8^	10.26 x 10^6^ ± 1.3 x 10^6^	99.852 ± 0.218^b^
PUI	8.07 x 10^8^ ± 1.9 x 10^8^	5.57 x 10^5 ^± 3.9 x 10^5^	99.926 ± 0.049^b^
HEAT	1.36 x 10^9^ ± 5.1 x 10^8^	91.10 ± 121.85	99.999 ± 1.0 x 10^-5c^
EDD-HEAT	1.06 x 10^9^ ± 3.4 x 10^8^	0	100.00 ± 0.00^c^
END-HEAT	7.60 x 10^8^ ± 2.4 x 10^8^	0	100.00 ± 0.00^c^
PUI-HEAT	1.17 x 10^9^ ± 3.6 x 10^8^	0	100.00 ± 0.00^c^

The number of CFUs decreased significantly between S1 and S2 for the different groups (p < 0.001).

The percentage of bacteria reduction was very high for the CONV, END, EDD, PUI and H groups but significantly different from 100% (p < 0.05). The percentage of bacteria reduction was higher for END, EDD, PUI and H groups compared to group CONV (p < 0.001). Group H performed better than groups END, EDD and PUI (p < 0.05).

The CONV, END, EDD and PUI protocols were not able to completely remove bacteria in any of the 10 specimens. Five S2 samples were free of bacteria in Group H. All of the S2 samples in the groups in which the NaOCl was activated and heated were free of bacteria.

The percentage of bacteria reduction was statistically significant between groups in which NaOCl was not heated and the other groups (p < 0.001). The percentage of bacteria reduction was not statistically significant among H, END-H, EDD-H and PUI-H (p = 0.166).

## Discussion

This was the first study to evaluate ex vivo effect of intracanal heating of NaOCl on the elimination of bacteria.

The methodology of the present study and the choice of E. faecalis were similar to other studies [18, 19]. However, the results should be interpreted carefully considering the limitations associated with this conventional sampling technique [20].

Heating NaOCl, and its sonic and ultrasonic agitation enhance its decomposition [21]. Therefore, it was important to refresh NaOCl after each heating and/or agitation.

Increasing heating temperatures leads to a faster and greater antibacterial activity [7, 22-24]. For this reason, the System B was set at 150°C in the present study. The other reason was that when the System B was set at 150°C and an XF electric heat carrier was used, the temperature on the root surface did not exceed 47°C at which the periodontal tissues could be damaged [25].

The results showed that the different irrigating protocols reduced significantly the bacteria counts. However, as expected and similar to previous studies, conventional irrigation without agitation (Group CONV) was the least effective compared to the other groups in reducing the number of bacteria [19, 26].

In agreement with previous studies, sonic and ultrasonic agitation of NaOCl without heat were not successful in eliminating bacteria completely [19, 27]. NaOCl sonic agitation (groups END and EDD) and ultrasonic agitation (group PUI) without intracanal heating of NaOCl were similar in their ability to reduce the number of bacteria as demonstrated in a recent study [19].

Heating NaOCl reduced the number of bacteria significantly in in vitro studies [7, 23, 24]. Interestingly, group H was associated with a higher percentage of bacteria reduction in comparison to the groups without the use of heat (groups CONV, END, EDD and PUI). Therefore, it can be assumed that the application of intracanal heat was more effective than conventional irrigation and the irrigation protocols involving only sonic and ultrasonic agitation of NaOCl. This result could be explained by the enhanced efficiency of intracanal heated NaOCl associated with the increased antibacterial and pulpal dissolving abilities. It may be assumed that the increased dissolving ability enhanced the bacterial elimination by exposing more bacteria to the action of the heated NaOCl; hence, five samples out of 10 in Group H were bacteria-free after applying the corresponding irrigation protocol. None of the sample in the groups without heat activation was free of bacteria.

The disinfecting ability of sonic and ultrasonic agitation of NaOCl was similar when NaOCl was heated. All the S2 samples from the groups exposed to NaOCl agitation and intracanal heating of NaOCl were free of bacteria, in comparison to only five in heat-only group (group H). Although the difference in the percentage of bacteria reduction between the heat- and agitation-groups, and the heat-only group was not statistically significant, this finding could indicate the importance of sonic and ultrasonic agitation in association with heat application to render canals free of bacteria. The removal of the pulp tissue with sonic or ultrasonic activation of NaOCl prior to heat application would allow a better exposure of the bacteria to the heated NaOCl. A recent study showed that the ultrasonic agitation of intracanal heated NaOCl enhanced the penetration of NaOCl in the dentinal tubules and resulted in cleaner canals compared to PUI alone and conventional irrigation [10]. In another study, ultrasonic agitation of heated NaOCl rendered artificial canals cleaner than heating NaOCl alone [28].

## Conclusions

One of the main objectives of the root canal treatment is to eliminate bacteria. Irrigation during and the end of the canal preparation plays a major role in the elimination of infection. Sodium hypochlorite is widely used as an irritant due to its antibacterial and pulp dissolution abilities. Warming of NaOCl inside of the canal enhanced pulp tissue dissolution and resulted in cleaner canals. However, the antibacterial ability of intracanal heated NaOCl has never been evaluated. The present study showed that intracanal warming of 5.25% NaOCl could be considered as an alternative to conventional irrigation protocols with sonic or ultrasonic agitation. The agitation of 5.25% NaOCl sonically or ultrasonically followed by intracanal heated NaOCl is very promising with regards to the removal of infection. Further evaluation is needed.
